# Photosynthesis and Spatial Distribution of Surface Phytoplankton in the Yangtze Estuary and Adjacent Waters During Spring

**DOI:** 10.3390/biology14111628

**Published:** 2025-11-20

**Authors:** Haojie Hu, Jing Xia, Xiu Gao, Wenlian Huang, Jiuyi Pan, Zhi Chen, Ji Li

**Affiliations:** 1 State Key Laboratory of Submarine Geoscience, Key Laboratory of Polar Ecosystem and Climate Change, Ministry of Education, Shanghai Key Laboratory of Polar Life and Environment Sciences, School of Oceanography, Shanghai Jiao Tong University, Shanghai 200030, China; 2 Shenzhen Public Platform for Screening and Application of Marine Microbial Resources, Institute for Ocean Engineering, Shenzhen International Graduate School, Tsinghua University, Shenzhen 440305, China; 3 Royal School of Mines, Imperial College London, South Kensington Campus, London SW7 2AZ, UK

**Keywords:** photosynthesis, chlorophyll fluorescence, CHEMTAX, estuary, environmental factors

## Abstract

Chlorophyll fluorescence provides direct insights into the physiological status of algae, contributing to the understanding of the marine carbon cycle. However, in situ measurements of phytoplankton photosynthetic physiology remain relatively scarce in the Yangtze River Estuary (YRE), an ecosystem under intense anthropogenic pressure. Consequently, quantifying composition and physiological status variations of phytoplankton in this region is critical for understanding their ecological functions and responses. Spring cruise revealed that the warm and high-salinity oceanic region exhibited a greater abundance of dinoflagellates and chrysophytes, while the lower-salinity plume region was characterized by higher abundances of chlorophytes and cryptophytes. Diatoms dominated across all regions. The highest chlorophyll *a* concentration (6.3 μg/L) was observed in the oceanic region. Chlorophyll fluorescence indicated that the warm offshore community was more active, suggesting favorable phytoplankton growth. As temperature decreased and seawater mixed, the maximum relative electron transport rate (rETRmax) and the minimal saturated light intensity (*I*_k_) decreased, yet the overall community remained healthy. Despite the plume delivering abundant nutrients, phytoplankton activity was relatively low due to the cold spring water temperature. This study will provide a foundation for understanding phytoplankton dynamics under anthropogenic influences in the YRE and the adjacent East China Sea, supporting algal bloom monitoring and early warning efforts.

## Simple Summary

Phytoplankton are key players in marine ecosystems and the carbon cycle, yet their in situ physiological responses to environmental gradients remain underexplored. This study investigated the surface phytoplankton communities and photosynthetic activity in spring across the Yangtze River Estuary and the adjacent East China Sea. By combining chlorophyll fluorescence measurements and pigment analysis, we found distinct spatial differences driven by temperature and salinity. Diatoms dominated throughout, while chlorophytes and cryptophytes thrived in low-salinity plume waters, and dinoflagellates and chrysophytes were more abundant offshore. Phytoplankton in warmer, high-salinity waters exhibited stronger photosynthetic activity, while colder coastal waters showed reduced efficiency despite nutrient enrichment. These findings highlight how environmental gradients shape phytoplankton physiology and distribution, and suggest that low spring temperatures may limit bloom development even when nutrients are abundant. This work improves our understanding of estuarine algal dynamics and supports early warning efforts for harmful algal blooms in rapidly changing coastal ecosystems.

## 1. Introduction

Estuarine ecosystems, characterized by high productivity, rich biodiversity, complex biogeochemical processes, and strong anthropogenic influences, have long been focal areas of public concern and scientific research [1,2,3,4]. World-renowned estuaries such as Chesapeake Bay, San Francisco Bay, the Gulf of Mexico, and the Pearl River Estuary have had their physical, chemical, and biological processes extensively studied [5,6,7,8,9,10,11]. The Yangtze River (Changjiang River), spanning over 6300 km, is China’s largest river and the world’s third largest, with an annual runoff of approximately 905.1 billion m^3^ and transporting about 106 million tons of sediment [12]. It traverses 11 provinces and municipalities in China, nurturing world-class wetland nature reserves and the renowned Yangtze River Delta urban agglomeration. The Yangtze River Estuary (YRE), serving as a crucial gateway connecting to the East China Sea (ECS), is also an ideal site for studying biogeochemical cycling processes [13,14]. On one hand, the YRE is situated at the confluence of the ECS and the Yellow Sea (YS), influenced by complex ocean currents that form a series of fronts, resulting in broad environmental gradients and intricate ecological processes [15,16,17]. The major currents influencing the YRE include the Changjiang Diluted Water (CDW), the ECS Coastal Current, the YS Coastal Current, the Taiwan Warm Current, and a branch of the Kuroshio Current [15,16,18]. On the other hand, the Yangtze River basin supports a population exceeding 400 million people and hosts extensive agricultural, industrial, and urban development [19]. Since the 1980s, nitrogen and phosphorus discharges into the YRE have continuously increased, leading to water eutrophication [15,16,20]. Numerous researchers have conducted sustained observations on the hydrological characteristics, water quality changes, and seawater stoichiometry in the YRE and the adjacent ECS [15,19,21,22]. Concurrently, field investigations and modeling studies have been conducted on the phytoplankton community’s composition, seasonal dynamics, primary production, and algal blooms [14,23,24,25]. However, studies on the in situ physiology of phytoplankton remain scarce. Investigating the distribution characteristics of phytoplankton photosynthesis in estuaries can aid in predicting blooms’ occurrence [26], understanding the primary productivity dynamics, and elucidating marine carbon cycling processes [27].

The assessment of the physiological status in marine phytoplankton is often achieved through in situ measurements of chlorophyll fluorescence, reflecting photosynthetic physiological traits [5]. Photosynthesis, being the most crucial biochemical process in plants, directly gauges carbon assimilation [27]. Chlorophyll fluorescence in phytoplankton often exhibits significant variations across different marine regions [5,28,29,30]. The maximum photochemical quantum yield of Photosystem II (*Fv*/*Fm*) is one of the most direct and common parameters for assessing the physiological status of algae [31]. Furthermore, the initial slope (α), the maximum relative electron transport rate (rETRmax), and the minimal saturated light intensity (*I*_k_) reflect light utilization efficiency, electron transport capacity, and adaptation to high light intensity, respectively [32]. Under conditions of sufficient nutrients, suitable light, and optimal temperature, phytoplankton typically exhibit higher *Fv*/*Fm* (>0.6) and higher electron transport rates [5,31]. Conversely, under nutrient limitation, suboptimal or excessive light, extreme heat or cold, or exposure to toxicants, algae often suffer from poor physiological status or even approach mortality, hindering efficient light energy utilization and transfer [5,30,31]. In summary, as a widely applied technique in aquatic ecosystems, the measurement of chlorophyll fluorescence parameters provides a rapid and accurate reflection of phytoplankton’s physiological status [28,29].

Although the physical transport, biogeochemical processes, community composition, and distribution in estuaries have been extensively studied, the spatial patterns of photosynthesis, a vital process, have rarely been documented [33]. Therefore, this study focuses on the in situ physiological status of surface phytoplankton in the YRE and the adjacent East China Sea during spring, utilizing chlorophyll fluorescence to characterize their photosynthetic features. Concurrently, pigment-based chemotaxonomy (CHEMTAX) is employed to understand the community composition and spatial dynamics. Thus, this research aims to explore the distribution patterns of phytoplankton communities and their photosynthetic characteristics along a series of environmental gradients within the YRE, providing fundamental data for the ecological management of estuarine and coastal ecosystems significantly impacted by intense human activities and disturbances.

## 2. Methods and Materials

### 2.1. Study Area and Sampling

**Figure 1:**
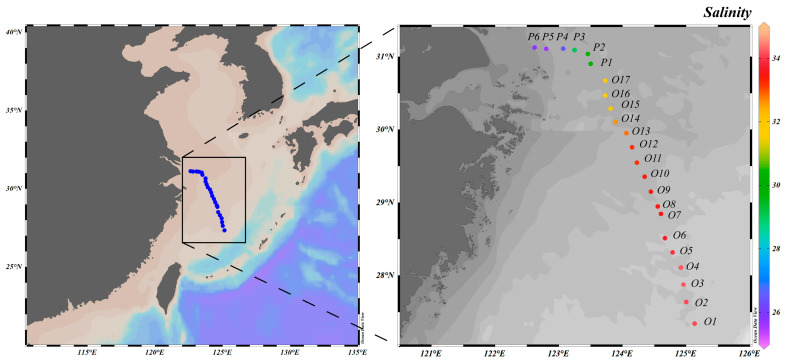
Map of the study area and sampling stations.

From 17 to 18 April 2018, an investigation was conducted aboard the R/V *Xuelong* in the YRE and the adjacent East China Sea (27°20′35.85″–31°7′40.52″ N, 122°37′4.36″–125°7′50.04″ E, Figure 1) on its way back from the Southern Ocean to Shanghai. Surface seawater (approximately 5 m depth) was continuously collected using the shipboard underway system. Subsequently, 1 L of the seawater sample was filtered under low pressure through glass fiber filters (25 mm diameter, Whatman, Maidstone, UK). The filters were stored in sterile bags, sealed to be light-sealed, and preserved at −80 °C until further analysis. The filtrate was stored in clean polyethylene bottles that had been pre-soaked in 10% hydrochloric acid for 48 h and kept at −20 °C.

Following the approach of Liu et al. (2016) [34], the study area encompassing the YRE and the adjacent East China Sea was divided into two regions: the plume region (salinity < 31) and the oceanic region (salinity ≥ 31).

### 2.2. Environmental Parameters Determination

To ensure the continuity and comparability of continuous environmental factors, daily data for sea surface temperature (SST) and sea surface salinity (SSS) were obtained from the National Marine Data Center, National Science & Technology Resource Sharing Service Platform of China (https://mds.nmdis.org.cn/, accessed on 10 May 2024), with a spatial resolution of 0.125. The suspended sediment concentration (SSC) was retrieved from the Level 1B (L1B) products of the Geostationary Ocean Color Imager (GOCI) satellite operated by the Korean Ocean Satellite Center (KOSC), via the online ocean remote sensing analysis platform SatCO2 (accessed on 29 June 2025) [35], at a spatial resolution of 0.006 × 0.006.

Concentrations of ammonium nitrogen (NH_4_^+^-N), phosphate phosphorus (PO_4_^3−^-P), and silicate silicon (SiO_3_^2−^-Si) were determined following the National Standard of the People’s Republic of China “Specifications for Oceanographic Survey-Part 4: Seawater Analysis” (GB 17378.4–2007), using the sodium hypobromite oxidation method, the molybdenum blue spectrophotometric method, and the silicomolybdenum blue method, respectively. Nitrate nitrogen (NO_3_^−^-N) was measured using a discrete analyzer (Seal AQ400, Seal Analytical, Mequon, WI, USA).

### 2.3. In Situ Quantification of Photosynthetic Characteristics

Fresh water samples collected on site were immediately transferred to dark bottles and subjected to a 15 min dark adaptation period. Subsequently, the chlorophyll fluorescence parameters of living algae were measured using a Phyto-PAM II phytoplankton analyzer (Heinz Walz, Effeltrich, Germany). The measurement sequence consisted of first determining the maximum photochemical quantum yield of Photosystem II (PSII) (*Fv*/*Fm*), followed by recording rapid light curves (RLCs) to α, rETRmax, and *I*_k_ [33].

### 2.4. Phytoplankton Community Composition Based on Pigment Analysis

Samples for pigment analysis were collected from representative stations covering both the oceanic region (stations O06–O10, O12–O17) and the plume region (stations P1–P6). The phytoplankton pigments concentrated on the filters were quantitatively analyzed for twelve specific pigments using high-performance liquid chromatography (HPLC) (Dionex UltiMate 3000 system, Thermo Fisher Scientific, Waltham, MA, USA). These pigments, used for calculating the phytoplankton community composition, included peridinin, 19′-butanoyloxyfucoxanthin, fucoxanthin, 19′-hexanoyloxyfucoxanthin, neoxanthin, prasinoxanthin, violaxanthin, alloxanthin, lutein, zeaxanthin, chlorophyll *a* (Chl *a*), and chlorophyll *b* (Chl *b*) [36]. The pigment-based matrix for phytoplankton community composition analysis referenced the methods established by Liu et al. (2012, 2016) [34,37].

### 2.5. Statistical Analysis and Visualization

Mapping and visualization were performed using Ocean Data View (version 5.6.7) [38]. Data statistics and plotting were conducted in R Studio (version 4.2.2). The “rstatix” package (version 0.7.2) was employed for Wilcoxon rank-sum tests comparing environmental factors and phytoplankton chlorophyll fluorescence parameters. The “psych” package (version 2.4.6) was used for Spearman’s rank correlation analyses. Principal component analysis (PCA) was carried out using the “FactoMineR” package (version 2.11). Data visualization was implemented with the “ggplot2” package (version 3.5.1). Missing environmental factor data were removed from the analyses. The functional relationships were quantified using fifth-order polynomial regression models using the poly function in R. Pigment-based phytoplankton community composition was calculated using CHEMTAX [39] in the R Studio environment with the “BEC” package (version 2.2.0) and the “limSolve” package (version 1.5.7.1).

## 3. Results

### 3.1. Basic Environmental Parameters in the YRE and the Adjacent East China Sea

A spring cruise was conducted along the YRE (YRE) and the adjacent East China Sea (Figure 1, Table 1). Significant environmental gradients were observed between the plume region and the oceanic zone. The results showed a continuous decrease in salinity from the open ocean toward the estuary, declining from 34.10 in the oceanic zone to 25.70 in the plume region (*p* < 0.05). Concurrently, sea surface temperature decreased from 22.8 °C in the oceanic zone to 14.6 °C in the plume region (*p* < 0.05).

In contrast, nutrient levels (including NH_4_^+^-N, PO_4_^3−^-P, SiO_3_^2−^-Si, and NO_3_^−^-N) exhibited a fluctuating increase from the open ocean toward the coast. Except for PO_4_^3−^-P, all other nutrients showed significant regional differences (*p* < 0.05), reflecting distinct environmental gradients. NH_4_^+^-N and NO_3_^−^-N concentrations increased significantly in coastal waters, with NO_3_^−^-N reaching its peak value of 21.30 μM. SiO_3_^2−^-Si ranged from 1.01 to 7.93 μM in the oceanic zone, while concentrations in the plume region reached 5.17–31.11 μM. Notably, PO_4_^3−^-P concentrations in the YRE and coastal waters were remarkably low (<0.11 μM), indicating pronounced phosphorus limitation. SSC concentration displayed an initial decrease followed by an increase. Elevated SSC levels (>15 mg/L) were observed to peak at 29.70 mg/L (Table 1).

### 3.2. Distribution of Phytoplankton Photosynthetic Parameters in the YRE and the Adjacent East China Sea

**Figure 2:**
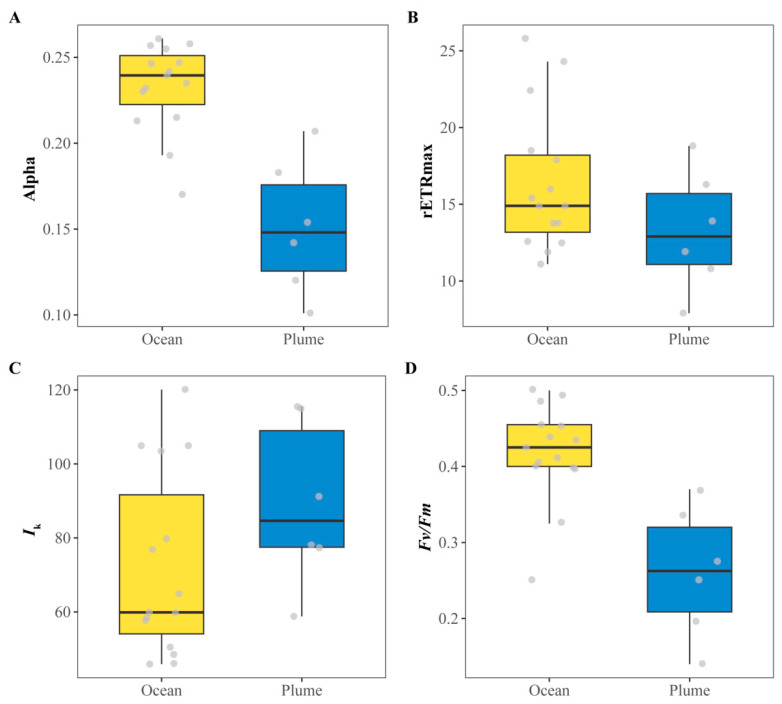
Distribution of phytoplankton chlorophyll fluorescence parameters in the Yangtze River estuary and the adjacent East China Sea. (**A**) The initial slope (Alpha). (**B**) The maximum relative electron transport rate (rETRmax). (**C**) The minimal saturated light intensity (*I*_k_). (**D**) The maximum photochemical quantum yield of photosystem II (*Fv*/*Fm*). The grey dots represent the sample points (*n* = 15 in the oceanic zone and *n* = 6 in the plume zone).

The in situ chlorophyll fluorescence parameters of phytoplankton in the study area are shown in Figure 2 and Table 2. Overall, the photosynthetic characteristics of phytoplankton exhibited distinct patterns between the oceanic zone and the plume region. Across the study area, phytoplankton photosynthetic parameters generally displayed a declining trend with fluctuations. *Fv*/*Fm* and α showed similar distribution patterns, with significant differences between the plume region and the oceanic zone (*p* < 0.05). Specifically, lower values were observed in some stations in the outer oceanic zone and the inner plume region, while the central area exhibited a more active physiological status. In the oceanic zone, *Fv*/*Fm* remained around 0.4, α averaged 0.23, and rETRmax peaked at 25.80, indicating healthy growth and an active physiological status. In contrast, within the plume region, *Fv*/*Fm* and α decreased to 0.26 and 0.15, respectively, while rETRmax did not exceed 20. The distribution patterns of rETRmax and *I*_k_ were also similar: higher values occurred in the outer oceanic zone, followed by a sharp decline and then a slow, fluctuating recovery.

**Figure 3:**
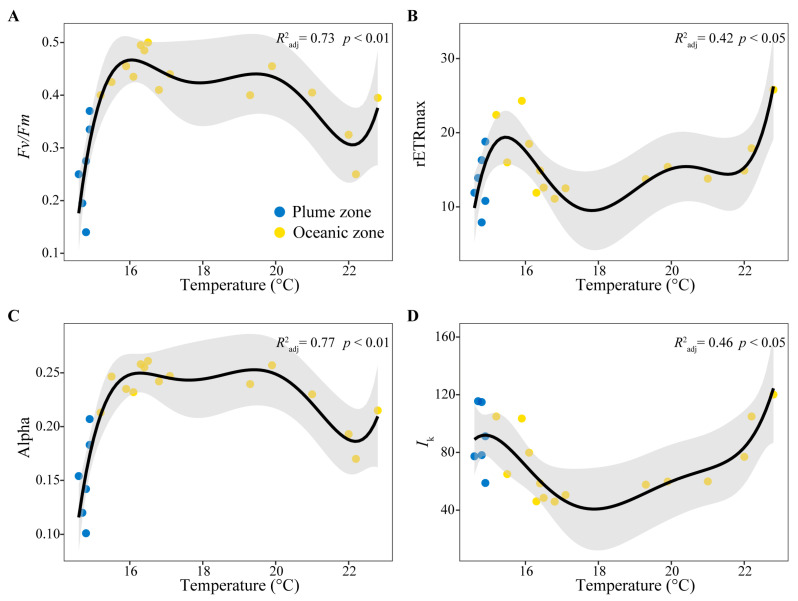

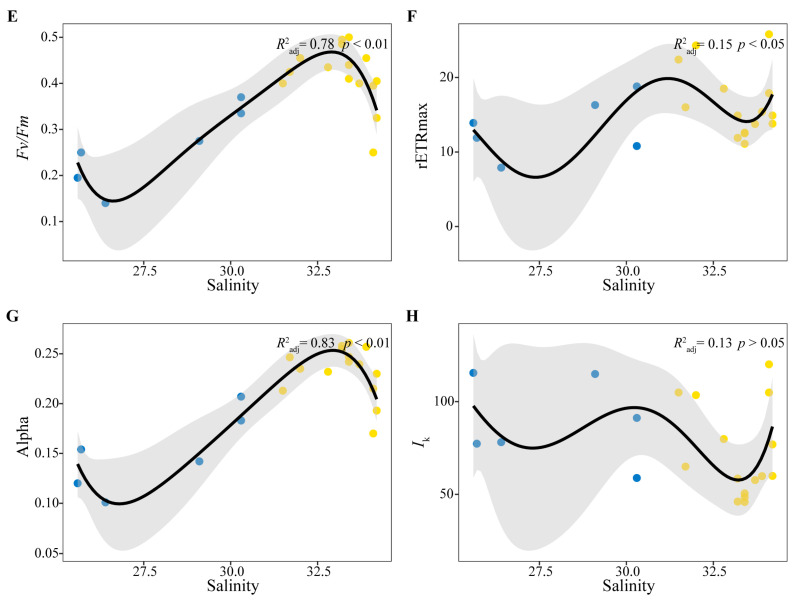
Smooth-fit curve based on a fifth-order polynomial regression model showing *Fv*/*Fm*, rETRmax, α and *I*_k_ as a function of temperature (**A–D**) and salinity (**E–H**) with *p* value and adjusted *R*^2^.

Temperature and salinity, as fundamental environmental factors in the ocean, directly influenced the physiological status of phytoplankton (Figure 3). As the distance from the coast decreased and sea surface temperature (SST) declined, phytoplankton *Fv*/*Fm* remained relatively high (–0.4) at SSTs above 15 °C. However, as temperatures decreased further within the plume region, *Fv*/*Fm* rapidly declined below 0.4, reaching a minimum of only 0.14. Moreover, α exhibited a similar distribution: most stations in the warmer open ocean maintained higher values (>0.2), while in nearshore areas, α decreased rapidly below 0.2 as temperatures dropped. In contrast, rETRmax and *I*_k_ showed an initial increase followed by a decrease with declining temperature, peaking in the area where the plume and open ocean waters converged.

Regarding salinity, *Fv*/*Fm* and α were also significantly influenced (Figure 3). As the distance to the estuary mouth decreased, *Fv*/*Fm* and α exhibited an S-shaped pattern in response to decreasing salinity: higher photosynthetic activity was observed in the high-salinity open ocean, followed by a recovery in areas closer to the estuary mouth.

### 3.3. Phytoplankton Community Composition in Representative Areas

**Figure 4:**
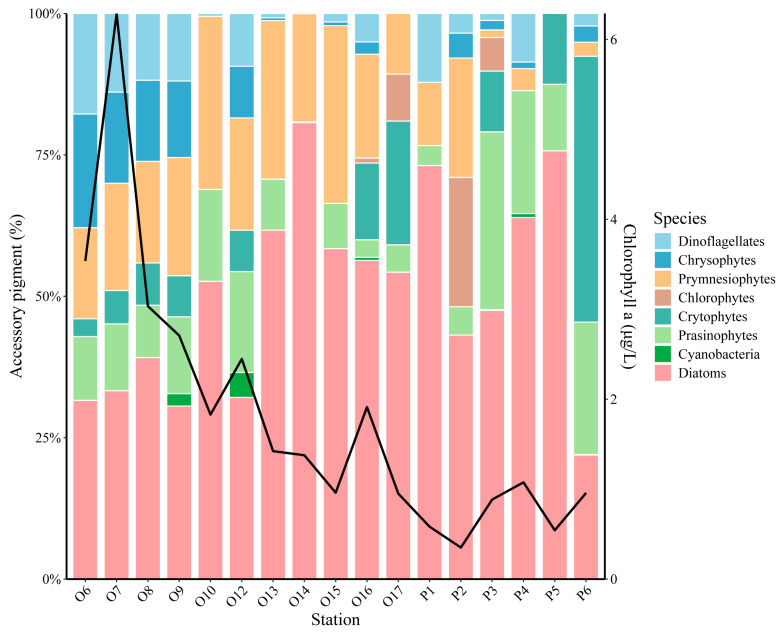
Phytoplankton community composition at representative areas and stations based on pigment analysis.

Pigment data obtained via HPLC were used to analyze the community composition at representative areas and stations within the YRE (YRE) and the adjacent East China Sea (Figure 4). Diatoms, present at all stations and contributing 21–80% of the total Chl *a*, were the dominant taxonomic group throughout the study area. Prymnesiophytes were the second most abundant group, found at the majority of stations. They contributed approximately 21% of Chl *a* in the oceanic zone, but their abundance decreased significantly within the plume region, averaging only about 6%. Similarly, dinoflagellates, chrysophytes, and cyanobacteria exhibited higher relative abundances in the warmer, higher-salinity waters of the outer oceanic zone. Their contributions gradually declined within the lower-salinity plume zegion. Dinoflagellates and chrysophytes were present at most stations, while cyanobacteria were only detected at some stations. Conversely, prasinophytes, chlorophytes, and cryptophytes were more prevalent within the cooler, lower-salinity waters of the plume region, contributing an average of 5–16% to the total Chl *a*.

Notably, Chl *a* concentration reached a peak of 6.3 μg/L in the warmer outer oceanic zone and subsequently declined gradually with decreasing temperature and salinity to an average of 0.8 μg/L within the plume region (Figure 3).

### 3.4. Correlation Analysis Between Phytoplankton Photosynthetic Characteristics and Environmental Factors

Table 3 presents the correlations between phytoplankton photosynthetic characteristics and environmental factors. *Fv*/*Fm* and α showed significant negative correlations with NH_4_^+^-N levels (*p* < 0.05). Conversely, α exhibited significant positive correlations with both salinity and temperature (*p* < 0.05).

### 3.5. Principal Component Analysis (PCA)

**Figure 5:**
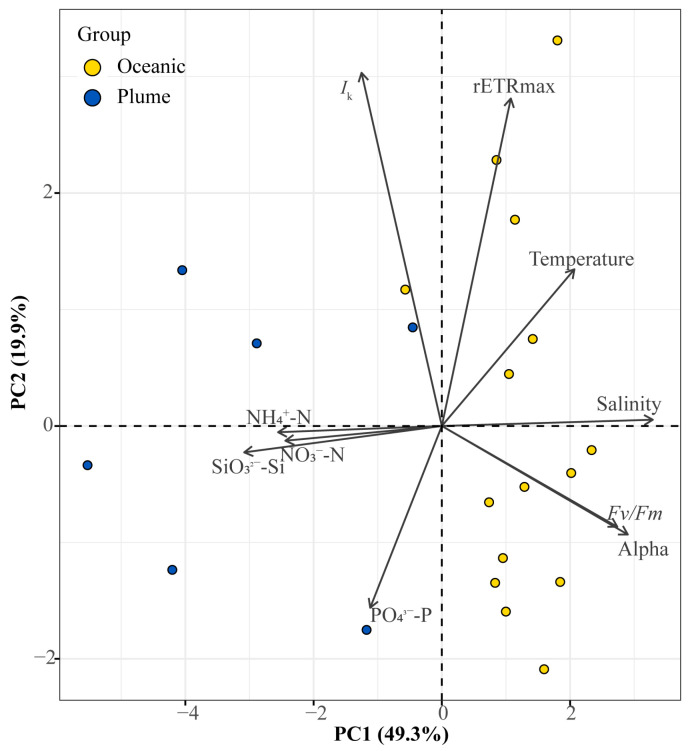
Principal component analysis (PCA) of factors influencing phytoplankton chlorophyll fluorescence parameters in the Yangtze River estuary and the adjacent East China Sea.

As shown in Figure 5, the cumulative contribution of the first principal component (PC1, 49.3%) and the second principal component (PC2, 19.9%) reached 69.2%. Salinity, *Fv*/*Fm*, α, and temperature were major contributors to the positive direction of PC1, while SiO_3_^2−^-Si and NH_4_^+^-N contributed significantly to the opposite direction. rETRmax and *I*_k_ were the primary contributors to PC2. Phytoplankton communities from the oceanic zone and the plume region showed clear separation. In the warmer, high-salinity oceanic zone, phytoplankton exhibited higher values of *Fv*/*Fm*, α, rETRmax, and *I*_k_, indicating a more favorable physiological status. However, within the plume region and at stations experiencing sharp environmental gradients characterized by lower temperature and salinity, phytoplankton displayed relatively lower light utilization efficiency (reflected by *Fv*/*Fm* and α), electron transport rates (rETRmax), and adaptation to high light (*I*_k_). Although nutrient inputs from the YRE supplied abundant nitrogen and silicon, the phytoplankton community composition shifted significantly (Figure 4), and cells experienced a degree of physiological stress.

## 4. Discussion

### 4.1. Spatiotemporal Dynamics of Phytoplankton Communities Shaped by Environmental Gradients

Compared with the open ocean and deep sea, estuaries exhibit more complex environmental gradients [11,40]. The intricate frontal systems and currents within the YRE create pronounced environmental gradients and highly variable planktonic spatial patterns [41]. Salinity is a key determinant of species distribution [5,42,43,44]. For instance, dinoflagellates thrive in polyhaline and euhaline waters, while chlorophytes and cryptophytes prefer fresher conditions (Figure 4). In contrast, diatoms appear more euryhaline, dominating across the entire salinity spectrum (Figure 4). This distribution pattern aligns with the observations by Shen et al. (2025) [44]. Similar salinity-dependent species distributions have also been documented in the Pearl River Estuary and the South China Sea [5]. Indeed, strong phytoplankton community differentiation along environmental gradients from estuaries to continental shelves is a widespread phenomenon in many coastal ecosystems globally [45]. Representative diatoms in the YRE include species of *Skeletonema*, notably *Skeletonema costatum*, which is a common dominant species year round [24,44,46,47]. Additionally, *Coscinodiscus* spp. are also prevalent, showing greater dominance during winter and spring [24]. Temperature is another critical factor governing algal distribution [44]. Chl *a* concentrations were relatively higher in the warmer oceanic waters; however, concentrations declined as temperatures decreased towards the coast, falling below 15 °C (Figure 4).

Nutrient levels also significantly influence phytoplankton’s abundance and diversity. Generally, high concentrations of terrestrially derived nutrients often trigger rapid, substantial phytoplankton growth and even algal blooms [48,49]. However, in this study, despite elevated levels of NH_4_^+^-N, SiO_3_^2−^-Si, and NO_3_^−^-N in the nearshore plume region due to terrestrial inputs, Chl *a* concentrations decreased instead of increasing. This counterintuitive pattern may be attributed to the still-low spring sea surface temperatures limiting phytoplankton growth [44]. Most phytoplankton grow optimally between 18 and 35 °C, with diatoms being relatively more cold-tolerant [50]. In the outer, lower-nutrient waters of the YRE during spring, average sea temperatures reached 18 °C, likely influenced by the Kuroshio Current, providing suitable conditions for phytoplankton’s growth and reproduction.

Dominant taxa in the East China Sea, such as the diatom *S. costatum* and the dinoflagellate *Prorocentrum donghaiense*, frequently form algal blooms [41,51,52]. The highest Chl *a* concentration measured in this study occurred at station O7, reaching 6.3 μg/L, with the diatom contribution peaking at 80%, indicating a trend toward significant algal proliferation. Small-scale patches of algal blooms begin developing in the East China Sea as early as April, typically peaking in July and dissipating by October [14]. The YRE is predominantly characterized by phosphorus limitation (Table 1). With the continuous discharge of terrestrially derived nitrogen, the N/P ratio in the estuary is further increasing. This shift promotes a transition in bloom-forming species from diatoms to dinoflagellates, elevating the risk of outbreaks involving toxic species [15,19,23,53]. Therefore, implementing long-term and systematic monitoring cruises is essential, both for early warning of bloom occurrences and for tracking the dynamics of dominant species [54].

It is noteworthy that the CHEMTAX approach employed in this study may carry certain limitations in its estimation of the phytoplankton community composition. These potential inaccuracies likely stem from inherent uncertainties associated with the method [55,56]. Comparatively, CHEMTAX-derived taxonomic groups are often less resolved than those obtained via high-throughput sequencing. Pigments shared across multiple species are difficult to accurately assign, potentially leading to erroneous estimates for certain phytoplankton groups [57]. Although Prochlorococcus is not a major chlorophyll contributor in the nutrient-rich Yangtze River Estuary, the absence of its diagnostic pigment, divinyl chlorophyll a (DV-Chl a), may lead to an underestimation of the entire cyanobacterial community. Furthermore, peridinin is not universally present in all dinoflagellates and exhibits significant interspecies variability. These factors collectively introduce biases into the phytoplankton community composition derived by CHEMTAX. Therefore, the initial matrix should be further optimized in the future. In recent years, multi-method approaches have been increasingly employed in phytoplankton community studies. However, interpreting the results requires caution. For instance, Xia et al. (2024) [5] and Xu et al. (2023) [58] conducted a comparative analysis of phytoplankton in the Pearl River Estuary using both CHEMTAX and 18S rRNA sequencing. Their results indicated that dinoflagellate and cryptophyte abundance might be misestimated by 18S rRNA due to their different gene copy numbers. Beyond these limitations, microscopy-based morphological identification remains a widely adopted and historically established classical method. However, its accuracy is heavily dependent on the taxonomic expertise of the researcher. Consequently, integrating multiple methodological approaches represents a superior strategy, as it leverages the respective strengths of each technique to provide a more comprehensive understanding of phytoplankton communities.

### 4.2. Factors Influencing the Distribution of Phytoplankton Photosynthetic Characteristics

Beyond phytoplankton community dynamics, their photosynthetic physiology is also profoundly influenced by environmental gradients. In the warmer, high-salinity oceanic waters, phytoplankton exhibited relatively higher values of *Fv*/*Fm*, α, rETRmax, and *I*_k_, indicating an active physiological state (Figure 2 and Figure 5). Generally, algae exhibit higher growth rates and more active photosynthesis within relatively optimal temperature ranges. However, once temperatures exceed or fall below this optimal range, both their growth and photosynthetic activity become inhibited [31]. In the plume region, a significant decline in photosynthetic activity was observed (Table 2). Furthermore, drastic environmental fluctuations may also lead to reductions in growth and photosynthetic performance. Within the central study area (stations O6–O12), coinciding with the convergence zone of the plume and oceanic waters, phytoplankton’s rETRmax and *I*_k_ decreased sharply in response to abrupt environmental shifts (Figure 2). This decline reflects reduced electron transport capacity and diminished tolerance to high light intensity. On one hand, abrupt shifts in salinity and temperature induced transient maladaptation in phytoplankton, resulting in fluctuating photosynthetic activity. On the other hand, low-salinity taxa typical of the plume region, such as chlorophytes and cryptophytes, exhibited reduced electron transport rates and diminished photoacclimation capacity due to their inability to rapidly adjust to sharply increasing salinity. Concurrently, the dominant phytoplankton groups shifted toward high-salinity-adapted taxa, notably dinoflagellates and chrysophytes (Figure 4). Collectively, these factors drove significant fluctuations in photosynthetic parameters within the central study area. Notably, values of *Fv*/*Fm* and α remained around 0.4 and 0.2, respectively (Figure 2), indicating that phytoplankton retained robust physiological health and sustained growth potential despite these environmental stressors. In the plume region, despite the supplementation of terrestrially derived nutrients, phytoplankton photosynthetic activity was relatively subdued under the cooler conditions. Light is also one of the important factors affecting the growth of phytoplankton [5]. At stations P4, P5, and P6, the reduced values of *Fv*/*Fm* (<0.3), α, and rETRmax may also be attributed to elevated SSC, which limits light availability. Nevertheless, the nutrient-rich Yangtze River plume plays an indispensable role in sustaining algal proliferation, particularly during summer [14,54]. Notably, the highest values of *Fv*/*Fm*, α, and rETRmax were observed at the leading edge of the plume–ocean convergence zone (Figure 2), signifying optimal photosynthetic performance. Although rETRmax and *I*_k_ were reduced in some areas, the generally high *Fv*/*Fm* values suggest that the algal cells retained robust photosynthetic potential but became relatively less active due to the sharp springtime gradients in temperature and salinity encountered in this region.

Li et al. (2023) [33] categorized estuarine phytoplankton on the basis of *Fv*/*Fm* values: stressed (<0.3), transitional (0.3–0.5), and bloom (>0.5). Wang et al. (2014) [26] proposed that *Fv*/*Fm* > 0.6 serves as a precursor to bloom initiation. In this study, the observed *Fv*/*Fm* values exceeded 0.4 across stations O4–O17, reaching up to 0.5. This indicates robust phytoplankton growth and the potential for continued proliferation. Despite sharp declines and fluctuations in rETRmax and *I*_k_ at some stations, the algal cells maintained physiological health, demonstrating sustained growth potential. Furthermore, suitable temperatures and elevated Chl *a* concentrations indicated the potential for bloom development. With rising water temperatures during summer, photosynthetically active phytoplankton are likely to further develop into algal blooms. Consequently, temperature emerges as a key driver of phytoplankton growth in the springtime Yangtze River Estuary (Figure 5). As noted earlier, diatoms dominate across all study regions due to their adaptation to cooler conditions. However, at stations with higher temperatures (O6–O10, O12), the abundance of other algal groups increased while diatoms’ dominance slightly declined. Beyond this taxonomic shift, peak values of photosynthetic physiological parameters and Chl *a* were consistently observed within the warmer oceanic region. The pronounced temperature gradient between the plume and oceanic regions during spring significantly influenced phytoplankton’s physiological status, driving distinct growth patterns. This aligns with Liu et al. (2017) [59], who also documented the substantial impact of temperature on phytoplankton communities in the YRE. Elevated water temperatures reduce CO_2_ solubility, leading to increased pH levels. Phytoplankton communities must physiologically adapt to these co-varying changes [59]. Moreover, warm seawater provides favorable conditions for bloom development. With rising summer temperatures, photosynthetically active phytoplankton populations tend to further proliferate into algal blooms.

Decades of persistent eutrophication in the YRE also provide favorable conditions for bloom outbreaks. On one hand, assessing phytoplankton’s physiological activity via chlorophyll fluorescence enables early detection of bloom precursors in the YRE, supplying essential data for long-term monitoring and management. On the other hand, this study employed a pigment-based chemotaxonomic approach to resolve phytoplankton communities, establishing a foundational dataset for tracking long-term phytoplankton dynamics in estuarine systems under climate change and anthropogenic pressures. Therefore, systematic long-term monitoring cruises are essential for estuaries, particularly those experiencing frequent anthropogenic disturbances, incorporating both phytoplankton community dynamics and photosynthetic physiology into the monitoring framework. Regardless, continuous monitoring of marine environmental changes and phytoplankton’s physiological status can effectively provide early warning of algal blooms. This study emphasizes that incorporating assessments of phytoplankton photosynthetic activity, alongside community composition and physiological status, is essential for bloom-prone estuaries like the YRE. This information is crucial for marine ecosystem management and provides a scientific basis for policy formulation.

## 5. Conclusions

This study conducted a comprehensive investigation into the community composition and in situ spatial distribution characteristics of phytoplankton photosynthesis in the YRE and the adjacent East China Sea during the spring. The results demonstrate that the extensive environmental gradients within the YRE drive significant spatial variations in phytoplankton’s distribution and photosynthetic activity. Diatoms emerged as the dominant phytoplankton group across the entire salinity spectrum, confirming their status as the prevalent functional group in both the YRE and nearshore ECS. Dinoflagellates and chrysophytes exhibited higher relative abundances in the high-salinity oceanic region, while chlorophytes and cryptophytes were more commonly distributed in the lower-salinity plume zone. Furthermore, the highest Chl *a* concentration was observed within the warmer oceanic region, coinciding with higher levels of phytoplankton photosynthetic activity. Although the plume region receives substantial inputs of terrigenous nutrients, the prevailing cooler spring temperatures resulted in lower Chl *a* concentrations compared with the oceanic region. Concurrently, these lower water temperatures constrained photosynthetic activity within the plume, although the phytoplankton assemblages retained a healthy physiological status. Despite the absence of major spring bloom events during the study period, sustained monitoring remains imperative. This research provides fundamental baseline data on phytoplankton dynamics within a highly anthropogenically disturbed estuary. The findings offer a necessary scientific foundation to inform future ecological management strategies and policy formulation for this critical coastal ecosystem.

## Figures and Tables

**Table 1:** Key environmental factors in the Yangtze River estuary and the adjacent East China Sea.

Environment Factors	Oceanic		Plume	
	Mean ± SD	Range	Mean ± SD	Range
Salinity	33.25 ± 0.89	31.50–34.10	27.90 ± 2.25	25.70–30.30
Temperature (°C)	18.20 ± 2.71	15.20–22.80	14.78 ± 0.12	14.60–14.90
NH_4_^+^-N (μM)	1.42 ± 0.28	1.08–1.93	1.92 ± 0.30	1.58–2.27
PO_4_^3−^-P (μM)	0.02 ± 0.02	<LD–0.06	0.04 ± 0.05	<LD–0.11
SiO_3_^2−^-Si (μM)	3.24 ± 1.97	1.01–7.93	14.61 ± 9.66	5.17–31.11
NO_3_^−^-N (μM)	1.02 ± 1.13	<LD–4.04	6.06 ± 8.00	1.40–21.30
SSC (mg/L)	3.43 ± 8.72	0.50–29.70	3.62 ± 6.54	0.60–16.9

Notes: LD means limit of detection.

**Table 2:** Phytoplankton photosynthetic physiological parameters across different regions.

Chlorophyll Fluorescence	*Fv*/*Fm*	Alpha	rETRmax	*I*_k_
Oceanic	0.42 ± 0.06	0.23 ± 0.03	16.38 ± 4.56	72.13 ± 24.86
Plume	0.26 ± 0.09	0.15 ± 0.04	13.27 ± 3.92	89.30 ± 22.56

**Table 3:** Spearman correlation analysis between phytoplankton photosynthetic characteristics and environmental factors.

Factors	Salinity	Temperature	NH_4_+-N	PO_4_^3−^-P	SiO_3_^2−^-Si	NO_3_^−^-N
*Fv*/*Fm*	0.37	0.34	−0.47 *	−0.09	−0.33	−0.16
Alpha	0.47 *	0.45 *	−0.52 *	−0.11	−0.38	−0.14
rETRmax	0.14	0.19	−0.14	−0.34	−0.39	−0.15
Ik	−0.30	−0.24	0.22	−0.15	0.06	0.11

Notes: “*” means *p* < 0.05.

## Data Availability

The original contributions presented in this study are included in the article. Further inquiries can be directed to the corresponding author.
